# Supplementary material for: Determination of Low Muscle Mass by Muscle Surface Index of the First Lumbar Vertebra Using Low-Dose Computed Tomography
Source: J Clin Med. 2022 Apr 26;11(9):2429. doi: 10.3390/jcm11092429 (PMC9103630; doi:10.3390/jcm11092429)

Determination of low muscle mass by muscle surface index of the first lumbar vertebra using low dose computed tomography.

Ping-Huai Wang[1,2], Chien-Hung Gow[1,3]*, Yen-Ling Chiu[4, 5, 6], Tien-Chi Li[7]

Affiliations:

[1]Division of Pulmonology, Department of Internal Medicine, Far Eastern Memorial Hospital, New Taipei City, Taiwan

[2]Department of Nursing, Asia Eastern University of Science and Technology, Taiwan

[3]Department of Healthcare Information and Management, Ming-Chuan University, Taoyuan, Taiwan

[4] Graduate Program in Biomedical Informatics, Yuan Ze University, Taoyuan, Taiwan

[5]Division of Nephrology, Department of Internal Medicine, Far Eastern Memorial Hospital, New Taipei City, Taiwan

[6] Graduate Institute of Clinical Medicine, National Taiwan University, Taipei, Taiwan

[7]Department of Radiology, Far Eastern Memorial Hospital, New Taipei City, Taiwan

***Correspondence:**

Chien-Hung Gow MD, Ph D

Division of Pulmonology, Department of Internal Medicine, Far Eastern Memorial Hospital, New Taipei City, Taiwan

Figure S1. Illustration of calculating L1MI by TomoVision SliceOmatic software

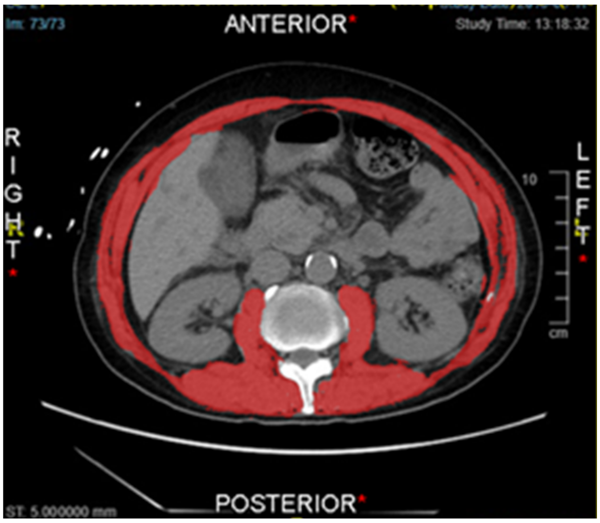

Figure S2. The scatter plots of L1MI and SMI (A) the study population (B) the COPD group

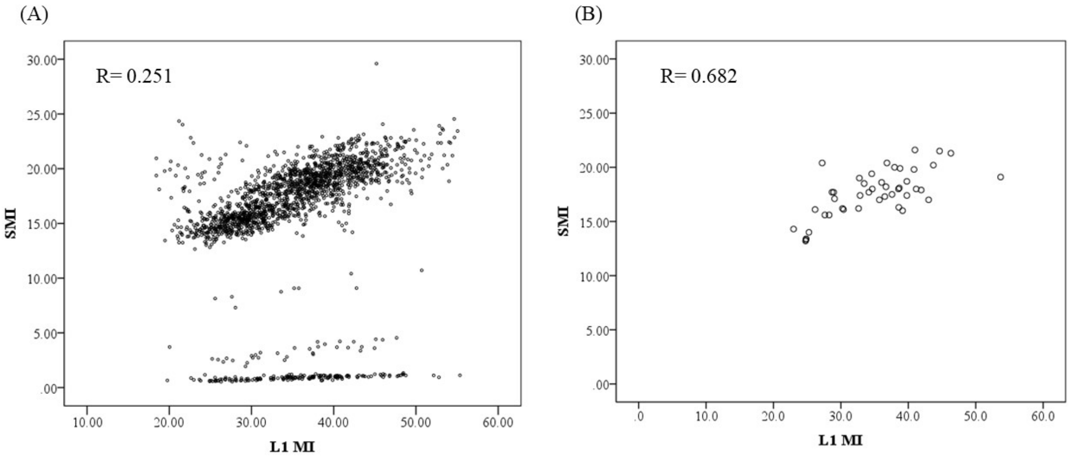

Supplement: Supplementary file 1 [file jcm-11-02429-s001.zip › jcm-1695126-supplementary.pdf]
